# Post-operative management following endoscopic sinus surgery in the UK: a survey of The British Rhinological Society

**DOI:** 10.1017/S0022215124002196

**Published:** 2025-09

**Authors:** Loic Hayois, Peter Andrews, Samuel C. Leong, Rishi Sharma, Neil Tan

**Affiliations:** 1 ENT, Royal Cornwall Hospitals NHS Trust, Truro, UK; 2 The Royal National ENT Hospital, University College London Hospitals, London, UK; 3 The Liverpool Head and Neck Centre, Liverpool, UK; 4 Addenbrookes Hospital, Cambridge, UK; 5 University of Exeter Medical School, Exeter, UK

**Keywords:** nasal polyps, rhinosinusitis, anti-bacterial agents, debridement, otolaryngologists, postoperative care, analgesia, nasal lavage, adrenal cortex hormones, surveys and questionnaires

## Abstract

**Objectives:**

The primary objective of our study was to survey ENT surgeons who perform functional endoscopic sinus surgery in the UK regarding their post-sinus surgery practices.

**Method:**

A 28-item questionnaire on post-functional endoscopic sinus surgery practices was distributed electronically to ENT UK members specialising in rhinology.

**Results:**

Ninety (90 per cent) surgeons prescribe saline nasal irrigation post-functional endoscopic sinus surgery but administration timing and methods vary. Following functional endoscopic sinus surgery, 17.7 per cent (*n* = 17) of respondents routinely prescribe antibiotics, whilst about a quarter (26.0 per cent, *n* = 25) do not prescribe antibiotics at all. The rest of the respondents only prescribe antibiotics in specific cases. Thirty-three (34.7 per cent) respondents do not prescribe oral steroids whilst most clinicians (83.9 per cent, *n* = 78) prescribe intranasal corticosteroids post-operatively.

**Conclusion:**

Our study highlights homogeneous, evidence-based practices post-functional endoscopic sinus surgery from UK-based specialists, specifically in the use of saline irrigation and intranasal corticosteroids. However, our cohort displayed significant heterogeneity regarding oral antibiotics, oral steroids, and other specific aspects of post-operative care.

## Introduction

With a prevalence of 11 per cent in Europe, chronic rhinosinusitis is dubbed the ‘silent epidemic’ due to its insidious nature.^1^ The initial management of chronic rhinosinusitis includes lifestyle modifications, followed by interventions such as intranasal saline and steroids and oral steroids. When medical treatments prove ineffective, functional endoscopic sinus surgery (FESS) is used to relieve obstruction of the common drainage pathways and restore sinus ventilation.^2^

Following FESS, various post-operative management strategies are advised to optimise chronic rhinosinusitis symptom resolution and prevent disease recurrence. This includes saline irrigation and intranasal corticosteroids, which boast substantial evidence supporting their effectiveness in the post-operative period.^3^ Conversely, specific post-operative measures, including oral corticosteroids, oral antibiotics, and nasal packing, lack comprehensive evidence to substantiate their widespread use and are considered as options only in recent guidelines.^2^ The paucity of data available in the literature concerning these post-operative measures has given rise to considerable variability in clinical practice among specialists, a phenomenon highlighted by Helman *et al*. in their 2019 survey of American Rhinologic Society members.^4^

The primary objective of our study was to survey ENT surgeons who perform FESS in the United Kingdom regarding their post-sinus surgery practices. We aim to compare these practices with existing evidence-based recommendations to shed light on the alignment of clinical practice in the UK with established best practices.

## Materials and methods

A 28-item questionnaire was developed using SurveyMonkey (San Mateo, CA). The questionnaire was trialled by three otolaryngologists for readability and appropriateness of content. This survey was electronically distributed to ENT UK members specialising in rhinology. Data were collected between September 2022 and April 2023 and respondents were assigned anonymous user IDs. Demographic characteristics of the respondents included level of seniority (consultant, post-Certificate of Completion of Training Fellow, registrar ST6-8 or ST3-5), completion of a rhinology fellowship and membership in ENT UK or the British Rhinological Society (BRS). Practice volume was assessed by the number of FESS cases performed per month excluding sinonasal oncology and skull base surgery.

The survey explored post-FESS practices including the use of saline irrigation, antibiotics, oral and intranasal steroids, nasal packing, analgesia, debridement of nasal crusts and clots, and follow up timings. Results were entered and analysed with Microsoft Excel 2016 (Microsoft Corp, Redmond, WA). Percentage response rates were calculated for each item based on the number of respondents for that specific item.

## Results

The survey was distributed to 486 members of the British Rhinological Society and completed by 100 surgeons (response rate 20.6 per cent) during the eight-month collection period. The response rate per question varied from 100 per cent (*n* = 100) to 43 per cent (*n* = 43) of survey respondents.

Most respondents were consultants (84.7 per cent, *n* = 72) while the remaining were post- Certificate of Completion of Training Fellows (3.5 per cent, *n* = 3), and registrars (ST3-5, 2.5 per cent, *n* = 2; ST6-8, 9.41 per cent, *n* = 8). 38 clinicians (38 per cent) had undertaken fellowship training in rhinology. Sixty-four surgeons (64 per cent) performed five or more FESS procedures per month while 26 (26 per cent) reported doing 2–4 cases per month and 10 (10 per cent) reported doing one case per month.

### Saline nasal irrigation

**Figure 1.**
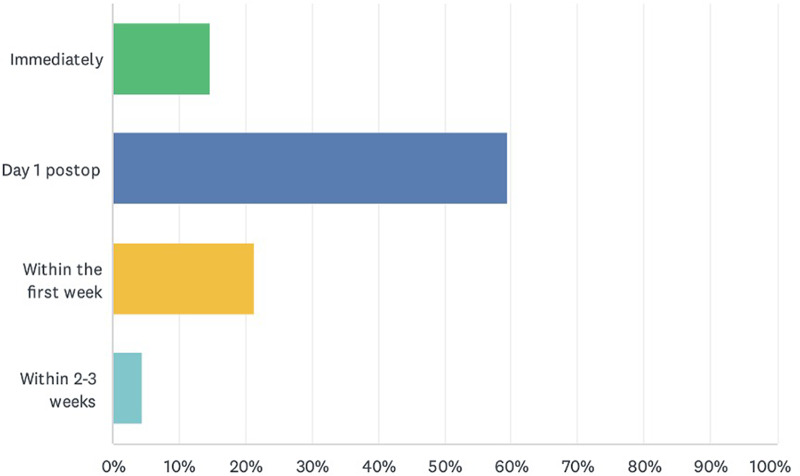
Timing of prescriptions for post-operative saline nasal irrigation; total respondents *n* = 89.

Ninety surgeons (90 per cent) prescribe saline nasal irrigation post-FESS. Around three-fourths (74.2 per cent, *n* = 66) of prescribers start saline nasal irrigation within 24 hours of FESS, while 21.4 per cent (*n* = 19) of them start it during the first week, and a minority (4.5 per cent, *n* = 4) initiate saline nasal irrigation within two to three weeks post-operatively (Figure 1).

Seventy-one respondents (79.8 per cent) prescribe high-volume, low-pressure saline irrigation and 28.1 per cent (*n* = 25) prescribe low-volume, low-pressure saline irrigation. Seven respondents (7.9 per cent) prescribe both. None of the respondents described using pulsating devices such as Neilmed (Santa Rosa, CA) Sinugator. Pre-prepared sachets such as Neilmed Sinus Rinse are the most popular form of nasal irrigation (65.1 per cent, *n* = 58), followed by patient-prepared solutions (e.g. cooking salt/bicarbonate) (49.4 per cent, *n* = 44) and pre-prepared syringes with saline vials (5.6 per cent, *n* = 5).

**Figure 2.**
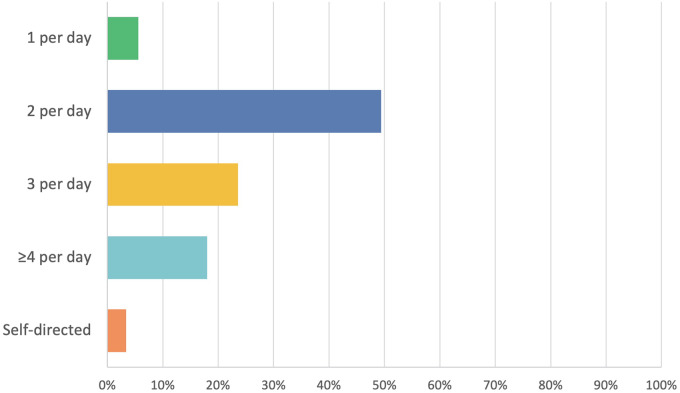
Frequency of nasal saline irrigation/spray; total respondents *n* = 89.

Figure 2 shows that about half (49.4 per cent, *n* = 44) of the surgeons advise their patients to use saline nasal irrigation or spray twice a day, whilst 23.6 per cent (*n* = 21) recommend it three times a day and 18.0 per cent (*n* = 16) at least four times a day. Only 5.6 per cent (*n* = 5) of prescribers recommend once a day saline irrigation or spray and 3.4 per cent (*n* = 3) of prescribers do not advise patients on a specific frequency.

### Antibiotics

**Figure 3.**
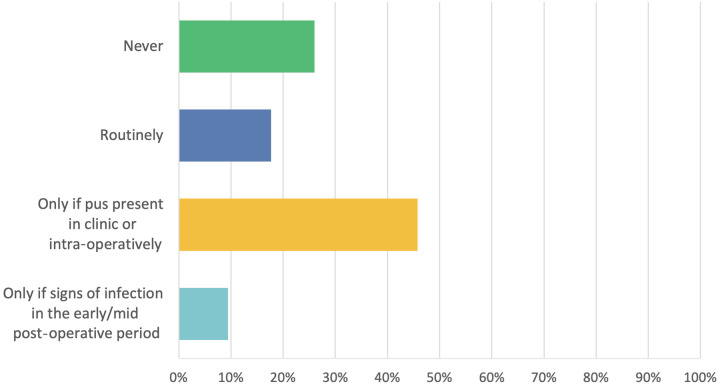
Antibiotics use in the immediate post-operative period; total respondents *n* = 95.

Following FESS, 17.7 per cent (*n* = 17) of respondents routinely prescribe antibiotics, whilst about a quarter (26.0 per cent, *n* = 25) do not prescribe any. Other respondents only prescribe antibiotics in specific cases, such as the evidence of intra-operative mucopus or previous demonstration of pus in clinic (45.8 per cent, *n* = 44), or signs of infection in the early or mid-post-operative period (9.4 per cent, *n* = 9) (Figure 3). One respondent prescribes antibiotics when a non-absorbable spacer is inserted intra-operatively.

Broad spectrum antibiotics, such as penicillin or cephalosporin, are the most widely used (64.3 per cent, *n* = 45), with a smaller proportion of clinicians (35.7 per cent, *n* = 25) preferring macrolides. Six respondents use doxycycline (8.6 per cent) and one respondent indicated using topical antibiotic.

### Steroid use

Thirty-three respondents (34.7 per cent) do not prescribe oral steroids post-operatively. In chronic rhinosinusitis patients with nasal polyps, 27.4 per cent (*n* = 26) of clinicians always prescribe oral steroids post-operatively, whilst 37.9 per cent (*n* = 36) only occasionally do. Conversely, in chronic rhinosinusitis patients without nasal polyps, only 5.3 per cent (*n* = 5) of respondents always prescribe oral steroids, whereas 12.6 per cent (*n* = 12) occasionally do.

Most clinicians (83.9 per cent, *n* = 78) prescribe intranasal corticosteroids in the post-operative period. Prescription timings vary with 56.0 per cent (*n* = 42) of clinicians starting intranasal corticosteroids within 24 hours of the operation, 17.3 per cent (*n* = 13) within the first week and 22.7 per cent (*n* = 17) delaying intranasal corticosteroids by two or three weeks post-operatively. Two respondents (2.7 per cent) start intranasal corticosteroids following completion of an oral steroid course and one respondent only starts this treatment following the first follow-up appointment if polyps are present.

Thirty-five respondents (46.7 per cent) use intranasal spray (e.g. fluticasone), 57.3 per cent (*n* = 43) apply intranasal drops (e.g. flixonase nasules) and 20.6 per cent (*n* = 15) utilise intranasal steroid irrigation (e.g. budesonide diluted in a saline douche).

**Figure 4.**
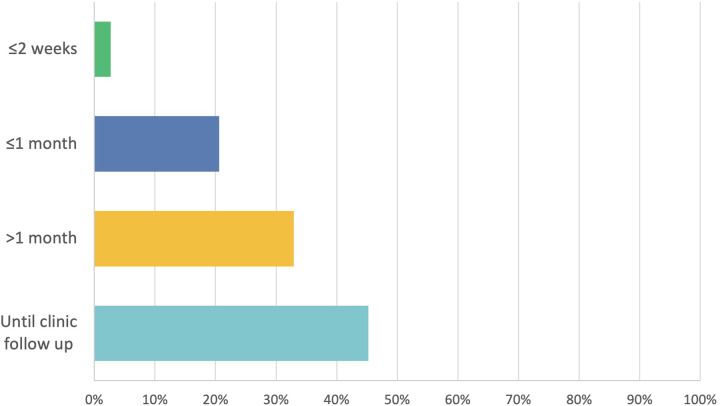
intranasal corticosteroids course length post-endoscopic sinus surgery; total respondents *n* = 73.

About half of clinicians (45.2 per cent, *n* = 33) do not set a course length for intranasal corticosteroids and these are given until follow up at least. Twenty-four clinicians (32.9 per cent) prescribe intranasal corticosteroids for more than one month whilst 20.6 per cent (*n* = 15) and 2.7 per cent (*n* = 2) prescribe intranasal corticosteroids for up to a month and up to two weeks, respectively (Figure 4).

### Analgesia

Paracetamol is the most widely used analgesic with 98.6 per cent (*n* = 71) of clinicians using it post-operatively. About half of clinicians (55.6 per cent, *n* = 40) routinely use non-steroidal anti-inflammatories and a smaller proportion of prescribers (22.2 per cent, *n* = 16) routinely use opioids post-operatively. Amongst respondents prescribing oral opioids, codeine is the most popular preparation post-operatively (61.5 per cent, *n* = 16). Fewer clinicians use dihydrocodeine (26.9 per cent, *n* = 7) or oral morphine (11.5 per cent, *n* = 3). Prescribing courses for oral opiates are 2–28 days with a mean of 7.0 days.

### Nasal packing, spacers and post-operative debridement

A large proportion (72.9 per cent, *n* = 62) of respondents indicated routinely (defined as >50 per cent of the time) using nasal packing post-FESS with dissolvable packs, such as Nasopore (Stryker, Kalamazoo MI), being more popular (64.7 per cent, *n* = 55) than removable packs, such as Merocele (Medronic, Minneapolis MN) (8.2 per cent, *n* = 7). Around a quarter of respondents (27.1 per cent, *n* = 23) do not routinely use any form of nasal packing post-FESS.

Most respondents (90.6 per cent, *n* = 77) do not regularly (>50 per cent of the time) use stents or spacers in the frontal recess or middle meatus after routine FESS. However, respondents who routinely use these devices favour inert stents such as silastic (8.2 per cent, *n* = 7) rather than drug-eluting stents such as Propel (Medtronic, Minneapolis MN) (1.2 per cent, *n* = 1).

**Figure 5.**
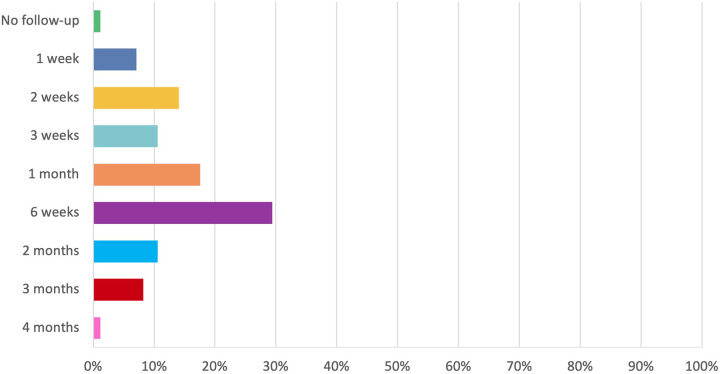
Follow up timing, post-endoscopic sinus surgery; total respondents *n* = 85.

Figure 5 highlights follow-up timing practices which are highly variable from no follow up, to review at four months post-operatively with the most common timings being one month (17.6 per cent, *n* = 15) and six weeks (29.4 per cent, *n* = 25). Thirty-four respondents (40.0 per cent) usually perform in-office debridement of nasal crusts and clots post-operatively. Twenty respondents (71.4 per cent) undertake debridement only once post-operatively, three respondents (10.7 per cent) twice, one respondent (3.6 per cent) three times and four respondents (14.3 per cent) do it as needed.

## Discussion

To our knowledge, this is the first study to report post-FESS management practices amongst UK-based ENT surgeons. Our findings suggest heterogeneity in the volume of sinus surgery performed with 36 clinicians completing fewer than five cases per month, including four respondents being registrars, 25 being consultants/post-Certificate of Completion of Training fellows and the rest not indicating their grade. The heterogeneity in FESS volumes between clinicians may highlight the differences between specialists in tertiary centres and generalists in secondary centres.

Most clinicians use post-operative saline irrigation as recommended by the International Consensus Statement on Allergy and Rhinology: Rhinosinusitis 2021.^2^ The International Consensus Statement on Allergy and Rhinology: Rhinosinusitis [2016] recommended that saline irrigation be started 24–48 hours after FESS.^5^ A proportion of our respondents do not follow these recommendations, with 14.6 per cent of them starting nasal irrigation immediately and 4.5 per cent starting it within 2–3 weeks. Some respondents (21.4 per cent) indicate that they start nasal saline irrigation within the first week, but this could fit the recommended 48-hour window or include delayed treatment initiation. Our findings contrast with a more recent survey by the American Rhinologic Society which showed significantly lower rates of saline irrigation use post-operatively.^4^

A large proportion of our respondents indicated using high-volume, low-pressure irrigation devices rather than low-volume, low-pressure devices. Although high-volume, low-pressure saline irrigation is strongly recommended by the medical management of chronic rhinosinusitis, the International Consensus Statement on Allergy and Rhinology: Rhinosinusitis 2021 guidelines have not specifically recommended this method of irrigation over any other methods post-FESS. Importantly, no respondents indicated using high-pressure devices which are currently supported by very limited evidence.^6^^–^^8^ Lastly, our survey shows heterogeneity in the frequency of nasal irrigation ranging from once a day to as often as the patient wants, which illustrates the lack of consensus in the literature.

The International Consensus Statement on Allergy and Rhinology: Rhinosinusitis 2021^2^ suggested that antibiotics should not be routinely prescribed but are an option post-operatively, which around half of our respondents adhere to by considering antibiotics only in specific circumstances. These numbers significantly contrast with American Rhinologic Society members surveyed in 2012 and 2019, where 79.6 per cent and 76.9 per cent routinely prescribed antibiotics in the immediate post-operative period.^4^^,^^9^ Similarly, Australian rhinologists surveyed in 2019 showed higher rates of post-operative antibiotic prescription than our cohort, with 68 per cent of them routinely using antimicrobials in patients who have had packing intraoperatively and 47.3 per cent of them prescribing antimicrobials in patients without packing.^10^ Our survey may illustrate a better adherence to judicious antibiotic prescribing in line with antibiotic stewardship.

Our respondents indicated a preference for broad spectrum antibiotics prescription over macrolides in the post-operative period. Randomised, controlled trials have shown that both broad spectrum antibiotics^11^ and macrolides^12^^,^^13^ tend to improve symptom and endoscopic scores in the FESS perioperative period, but with no statistical or clinical significance. Six respondents use doxycycline, which is an antibiotic that has not been specifically trialled in the FESS perioperative period. Overall, these results highlight the lack of consensus in the literature on the optimal agent and course length to be used.^14^

Despite the International Consensus Statement on Allergy and Rhinology: Rhinosinusitis 2021 guidelines,^2^ and the 2016 National Institute for Health and Care Excellence (NICE) Commissioning Guide^15^ for Chronic Rhinosinusitis strongly recommending the use of intranasal corticosteroids post-FESS, 16.1 per cent of respondents do not use intranasal corticosteroids post-operatively. Our survey did not explore the reasons behind this, but it would be interesting to understand why some respondents have not adopted intranasal corticosteroids as part of their routine post-operative care. There is however, no national or international recommendation on when treatment should be started post-operatively, and what preparation and course duration should be used. This lack of consensus is demonstrated by significant discrepancies in treatment initiation timings, types of intranasal corticosteroids preparation and course lengths.

Around a third of our respondent never prescribe systemic corticosteroids post-operatively which is a significantly higher proportion than American Academy of Otolaryngology–Head and Neck surgeons surveyed by Portela *et al*. (7.9 per cent)^9^ and Helamn *et al*. (22.1 per cent).^4^ In terms of oral corticosteroids prescriptions, our survey showed a wide range of practises with doses fluctuating between 20 mg and 60 mg of Prednisolone, duration of treatments ranging from single doses to one-month treatments and preparations also including prednisone and methylprednisolone. The lack of robust studies on oral corticosteroids post-FESS currently prevents standardised, evidence-based practises in this area of FESS post-operative care.

Our respondents almost unanimously prescribe paracetamol post-operatively which is a safe and efficacious analgesic post-FESS^16^^,^^17^ and is the first line analgesic recommended by the International Consensus Statement on Allergy and Rhinology: Rhinosinusitis 2021.^2^ About half of the respondents also use a non-steroidal anti-inflammatory drug (NSAID), an excellent choice of analgesic which, when used in combination with paracetamol, significantly reduces the need for opioids post-FESS.^18^ The oral opioid course average of 7.0 days amongst our respondents likely could be shortened as the literature suggests that patients usually require only a few doses following rhinological surgery.^19^^,^^20^

Almost three-quarters of respondents indicated routinely using nasal packing, which is recommended as an option by the International Consensus Statement on Allergy and Rhinology: Rhinosinusitis 2021.^2^ Although packing is not essential for intra-operative haemostasis and does not reduce the risk of post-operative epistaxis, there is some evidence, albeit limited, that packing reduces adhesion formation.^2^ Amongst our respondents, dissolvable packs are significantly more popular than removable packs. The evidence favours dissolvable packs rather than non-dissolvable packs in terms of patient comfort,^21^^,^^22^ but this advantage is not translated to better mucosal healing and surgical outcomes.^23^^,^^24^ Only one respondent routinely uses drug-eluting stents such as Propel despite the International Consensus Statement on Allergy and Rhinology: Rhinosinusitis 2021 highlighting that corticosteroid-eluting stents can be considered in the post-operative period.^2^ The cost, limited evidence and guarded International Consensus Statement on Allergy and Rhinology: Rhinosinusitis 2021 statements on the topic likely explain the negligible use of corticosteroid-eluting stents in our cohort.

Follow-up timings are heterogeneous between our respondents which fits the NICE Commissioning Guide for chronic rhinosinusitis recommending post-op reviews “tailored to the individual patient needs in terms of duration and frequency.”^15^ However, our questionnaire does not discriminate among different chronic rhinosinusitis subtypes and is therefore unable to identify the follow-up timings for specific patients cohorts. Only 40 per cent of respondents routinely perform post-operative debridement following FESS despite the International Consensus Statement on Allergy and Rhinology: Rhinosinusitis 2021 making a recommendation for this practice.^2^ A Cochrane review highlighted that there is little evidence to suggest that debridement improves disease severity or quality of life but there is low-quality evidence suggesting that this practice is associated with a lower risk of adhesions at three months follow up.^25^ These mixed findings, coupled with potential limitations of access to early follow-up appointments, may explain the poor rate of routine in-office debridement in our cohort.

There are several limitations to our study including heterogeneous response rates between questions which range between 66 and 100 per cent of respondents. Helman *et al*.^4^ in their survey of American Rhinologic Society members, highlighted significant differences in management strategies among the different chronic rhinosinusitis subtypes. Similarly, Ahmadzada *et al*. found significant differences in antibiotic prescription rates whether patients had packing intra-operatively or not.^10^ Other than the use of oral steroids, our survey does not disciminate among chronic rhinosinusitis subtypes, intra-operative management, or surgical indications.

- Certain aspects of post-operative care following endoscopic sinus surgery, such as intranasal saline irrigation, are backed by strong evidence, whilst there is a paucity of data regarding other measures, such as post-operative antibiotics
- Studies in the USA and Australia/New Zealand have shown that there is considerable variability in clinical practice among specialists
- This survey of 100 UK specialists regarding their post-operative care practices following functional endoscopic sinus surgery showed homogeneous, evidence-based, practices from UK-based specialists, specifically in the use of saline irrigation and intranasal corticosteroids
- However, there was significant variability in clinical practice regarding oral antibiotics, oral steroids, and more specific aspects of post-operative care such as formulation of drugs, doses and frequencies
- High-quality evidence is needed to provide more standardised care and inform the most effective post-endoscopic sinus surgery management

## Conclusion

Our study highlights homogeneous, evidence-based, practices post-FESS from UK-based specialists, specifically in the use of saline irrigation and intranasal corticosteroids. However, regarding oral antibiotics, oral steroids, and more specific aspects of post-operative care such as formulation of drugs, doses, and frequencies, our cohort displayed significant heterogeneity. In some cases, this heterogeneity may originate from a lack of education on the most up-to-date practices but in many areas of post-FESS care, there is a clear lack of high-quality evidence. High-quality evidence, in the form of robust randomised, controlled trials, is essential to inform the most effective post-FESS management and prevent recurrence of chronic rhinosinusitis, a disease that currently has a significant burden on patients and the healthcare system.

## Data Availability

Data available on request from the authors.
